# Periodic Motion Characteristics of a Magnetic Suspended Dual-Rotor System with Nonlinear Bearing Effects

**DOI:** 10.3390/s26144400

**Published:** 2026-07-10

**Authors:** Mingzheng Liu, Nianxian Wang, Xinyuan Chen, Yuan Xu, Yingjie Ding, Qiwei Wang

**Affiliations:** 1 School of Mechanical Engineering, Wuhan University of Science and Technology, No. 947, Heping Venue, Qingshan District, Wuhan 430081, China; liumz26@wust.edu.cn (M.L.); xuyuan@wust.edu.cn (Y.X.);; 2 Hubei Key Laboratory of Mechanical Transmission and Manufacturing Engineering, Wuhan University of Science and Technology, No. 947, Heping Venue, Qingshan District, Wuhan 430081, China

**Keywords:** magnetic suspended dual-rotor system, mass unbalance, nonlinear vibration, bifurcation

## Abstract

To investigate the nonlinear dynamic characteristics of magnetic suspended dual-rotor systems, this study examines periodic and quasi-periodic responses induced by bearing nonlinearities, including flux leakage and magnetic saturation effects. A nonlinear dynamic model is established using the finite element method, incorporating unbalance excitation and nonlinear bearing forces. A comprehensive parametric analysis is conducted to evaluate the effects of rotational speed, initial stiffness, and initial damping on the system’s dynamic responses and bifurcation behavior. The results reveal the occurrence of period-5 and quasi-periodic vibrations under nonlinear bearing conditions. In the quasi-periodic regime, low-frequency components dominate, and the force–current characteristics of the magnetic bearings spread over a wider band, reflecting a multi-valued force–current relationship. Furthermore, decreasing initial stiffness and increasing damping advance the onset of quasi-periodic responses and reduce the corresponding critical rotational speed. Notably, through real-time control adjustment, quasi-periodic motion can be converted into periodic motion, thereby distinguishing the system from conventional mechanically supported rotor systems. Experimental results obtained from a magnetic suspended dual-rotor test rig validate both the bearing-force model and the dynamic model, and further reveal periodic variations in system response under different speed ratios.

## 1. Introduction

In modern industry, dual-rotor configurations are widely employed to improve the efficiency and operational stability of aerospace and industrial gas turbines. A dual-rotor system supported in a contactless manner by active magnetic bearings (AMBs) is referred to as a magnetic suspended dual-rotor system (MSDS). Compared with conventional mechanically supported dual-rotor systems, MSDSs eliminate complex lubrication and sealing devices and enable tunable dynamic characteristics, thereby offering significant application potential [1].

Magnetic bearing–rotor systems are inherently nonlinear electromechanically coupled systems. Their stability plays a critical role in determining operational efficiency and service life, and has therefore attracted considerable attention. Existing studies have predominantly focused on disturbance control strategies, particularly the design and analysis of various controllers. Under these approaches, system stability is generally assessed by examining vibration responses in both the time and frequency domains. However, within this framework, periodic motions induced by nonlinearities—especially quasi-periodic oscillations—have not been systematically investigated. Moreover, the capability of control systems to regulate the evolution of and transitions between periodic motion states under varying operating conditions remains insufficiently explored. Consequently, both the fundamental characteristics of periodic motions and their controllable evolution mechanisms in magnetic bearing–rotor systems remain insufficiently understood.

In engineering practice, system faults and changes in bearing support conditions are often manifested as complex nonlinear vibration responses and the evolution of periodic motion characteristics. Therefore, a detailed investigation of rotor periodic motion is essential not only for understanding the evolution mechanisms of system stability, but also for providing vibration-response features for machinery fault diagnosis [2,3]. This is particularly relevant under complex operating conditions, such as those encountered in gas turbines, where significant load variations and strong dual-rotor coupling effects arise. Under such conditions, nonlinear factors in AMB systems—including flux leakage, magnetic saturation, and changes in the working floating position—become more pronounced [4]. These nonlinearities lead to strongly nonlinear support characteristics, thereby significantly influencing the periodic motion behavior of the rotor.

In recent years, the nonlinear dynamics of bearing–rotor systems have attracted significant research attention. Numerous studies have examined the effects of nonlinear disturbances, including rubbing [5,6,7,8], cracks [9], oil-film whirl [10], and strong base excitations [11,12], on rotor dynamic behavior. Yang et al. [6] and Wang et al. [13] developed finite element models of dual-rotor systems and analyzed their nonlinear dynamic responses in terms of frequency components and bifurcation characteristics. Poincaré maps and bifurcation diagrams have been widely used to characterize the rotor motion states. Regarding the nonlinear bearing characteristics, Jin et al. [14], Liu et al. [15], and Shun et al. [16,17] investigated nonlinear vibrations induced by rolling bearings, intermediate bearings, and oil-film damping, respectively. Their results demonstrated that rotor systems may exhibit jump phenomena, periodic motions, and chaotic motions, with dynamic states highly sensitive to system parameters. Similar phenomena have been observed in magnetic bearing–rotor systems; however, due to structural characteristics and the presence of active control, AMBs exhibit distinct nonlinear behaviors. Reported nonlinear factors include time-varying stiffness [18,19,20], amplifier nonlinearities [21], current saturation [22], magnetic saturation [23,24], and nonlinear contact forces in auxiliary bearings [25,26]. These studies demonstrate that the choice of operating parameters critically influences the nonlinear dynamic states of the system. Nevertheless, the combined effects of flux leakage and magnetic saturation on rotor dynamics have rarely been systematically investigated and thus warrant further study.

To investigate the influence of nonlinear factors, such as flux leakage and magnetic saturation, on magnetic bearing–rotor systems, establishing accurate and reliable magnetic bearing models is critical, and this topic has been addressed in numerous studies. Le et al. [27] and Yu et al. [28] developed dynamic magnetic circuit models to capture the effects of flux leakage in hybrid magnetic bearings. Zhang et al. [29] introduced a mathematical modeling approach based on precise segmentation of the magnetic field, enabling accurate evaluation of edge flux and leakage coefficients. To consider the combined effects of flux leakage, magnetic saturation, and edge flux, Wang et al. [30] proposed the nonlinear magnetic circuit method (NMCM) to construct an analytical bearing capacity model for AMBs. Experimental results confirmed that the NMCM maintained high accuracy across different air-gap lengths and rotor eccentricities. Wajnert et al. [31] and Wang S.Y. et al. [32] extended the NMCM to model magnetorheological clutches and hybrid bearings, respectively. The validity of these models was further supported by experimental verification. Overall, due to flux leakage and magnetic saturation, magnetic bearing support characteristics exhibit pronounced nonlinearity. Accurate nonlinear bearing models for AMBs can thus be effectively established using the NMCM.

In this study, an MSDS under PID control is investigated, and the NMCM is employed to characterize the nonlinear support behavior of magnetic bearings considering flux leakage, magnetic saturation, and changes in the working floating position. Furthermore, a finite element model of the dual-rotor system with unbalance excitation is established. The system equations are solved using the Newmark–β method, and the periodic motion characteristics are analyzed through trajectory diagrams, bifurcation diagrams, and Poincaré maps. The findings of this study provide useful insights into the modeling, parameter design, and control of periodic motion in magnetic bearing–rotor systems.

## 2. Dual-Rotor Model and Establishment of Dynamics Equations

**Figure 1:**
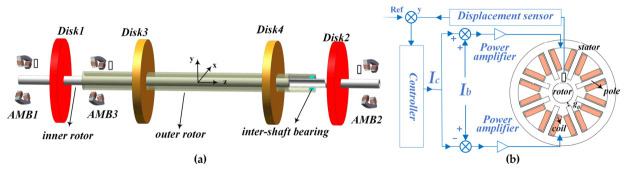
Schematic model of the MSDS. (**a**) Configuration of the dual-rotor system. (**b**) Control loop and structural configuration of the AMB.

In this study, an MSDS model is established, consisting of an inner rotor and an outer rotor, as shown in Figure 1a. The inner rotor represents the low-pressure rotor assembly, whereas the outer rotor represents the high-pressure rotor assembly and is arranged around the inner rotor as a hollow shaft. The two rotors are not rigidly connected and can therefore operate at different rotational speeds. The inter-shaft bearings permit relative rotation between the inner and outer rotors and transmit radial interaction forces, acting as the main coupling components of the dual-rotor system. The system is supported by three AMBs. Inner-rotor discs 1 and 2 represent the low-pressure compressor and turbine discs, respectively, while outer-rotor discs 3 and 4 represent the high-pressure compressor and turbine discs. Due to machining tolerances, mass unbalance exists in inner rotor disc 2 and outer rotor disc 4, as listed in Table 1. This study focuses on the radial vibration of the MSDS; therefore, torsional and axial vibrations are neglected to simplify the analysis. In addition, the effect of gravity is neglected.

### 2.1. Magnetic Bearing Modeling

The electromagnetic characteristics of the AMBs provide the basis for the dynamic analysis of the MSDS. In this subsection, a typical eight-pole radial AMB operating in differential mode is considered, and a nonlinear force model is established. The nomenclature is provided in Appendix A.

The magnetic pole pair in the y-direction is taken as an example, and its basic control loop is illustrated in Figure 1b. The controller adopts a PID strategy, where *I*_b_ and *I*_c_ denote the bias current and control current, respectively, and g_0_ represents the nominal air-gap length. The displacement sensor gain and the power amplifier gain are set to *A*_s_ = 800 and *A*_a_ = 0.6, respectively.

Based on the above AMB configuration, the nonlinear electromagnetic force model and the conventional linear electromagnetic force model are introduced in sequence to clarify the bearing-force modeling procedure.

**Figure 2:**
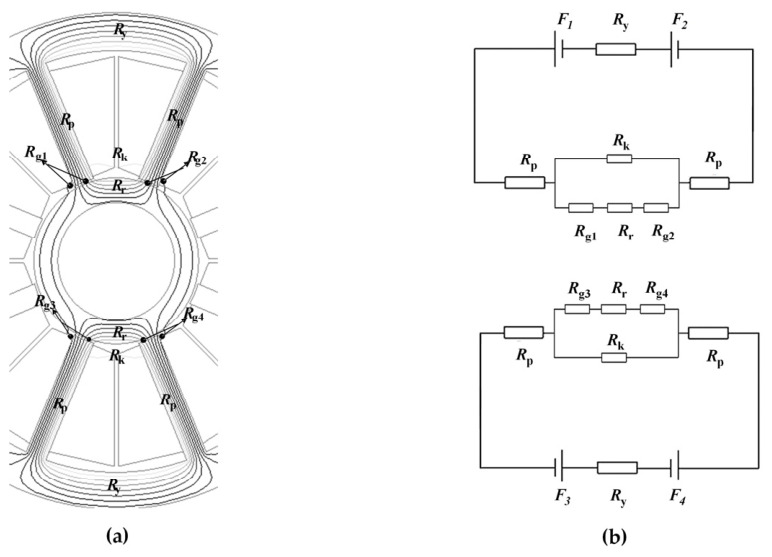
The nonlinear electromagnetic force modeling process using the NMCM. (**a**) Magnetic field division. (**b**) EMC for one pole pair based on the NMCM.

(a) Nonlinear electromagnetic force model: To quantify the influence of nonlinear factors such as flux leakage and magnetic saturation, following our group’s previous work [28], the magnetic flux regions are partitioned and analyzed using the NMCM method, as shown in Figure 2a. The reluctances of the four air gaps, leakage, yoke, poles, and rotor (denoted by *R*_g1_–*R*_g4_, *R*_k_, *R*_y_, *R*_p_, and *R*_r_, respectively) are derived. Based on these reluctances, the equivalent magnetic circuit (EMC) of a pole pair is established, as shown in Figure 2b. In the EMC, F1–F4 denote the magnetomotive force sources of the four poles. Then, the nonlinear electromagnetic force in the y-direction is obtained as follows:

(1) $$f_{y}=\frac{\cos\alpha }{\mu_{0}A_{p}}(\Phi_{1}^{2}-\Phi_{3}^{2}),$$

(2) $$\left\{\begin{array}{l} \Phi_{1}=\frac{N(I_{b}+I_{c})R_{k}}{\left(R_{g1}+R_{g2}+R_{r})(R_{k}+R_{y}+2R_{p})+R_{k}(R_{y}+2R_{p}\right)},\\ \Phi_{3}=\frac{N(I_{b}-I_{c})R_{k}}{\left(R_{g3}+R_{g4}+R_{r})(R_{k}+R_{y}+2R_{p})+R_{k}(R_{y}+2R_{p}\right)}, \end{array}\right.$$

where *α* denotes the angle between the centerlines of the pole and the pole pair (*α* = π/8); *μ*_0_ is the permeability of free space; *A*_p_ represents the cross-sectional area of the stator poles; *N* is the number of coil turns; and Φ_1_ and Φ_3_ denote the magnetic fluxes in the upper and lower magnetic branches of the pole pair associated with *R*_g1_–*R*_g2_ and *R*_g3_–*R*_g4_, respectively.

A nonlinear electromagnetic force model in the x-direction can be obtained using the same procedure.

(b) Linear electromagnetic force model: Flux leakage and magnetic saturation are neglected; specifically, *R*_y_, *R*_p_, *R*_r_, and *R*_k_ are neglected, and only the air-gap reluctance is considered. When the AMBs operate in the linear region, the electromagnetic force in a given direction (e.g., the y-direction) can be linearized as [23]:

(3) $$f_{y}=k_{x}x+k_{i}i,$$

(4) $$\left\{\begin{array}{l} k_{x}=-\frac{\mu_{0}A_{p}N^{2}I_{b}^{2}\cos\alpha }{g_{0}^{3}},\\ k_{i}=\frac{\mu_{0}A_{p}N^{2}I_{b}\cos\alpha }{g_{0}^{2}}, \end{array}\right.$$

where *k*_x_ and *k*_i_ are the displacement stiffness and current stiffness of the AMBs, respectively.

**Figure 3:**
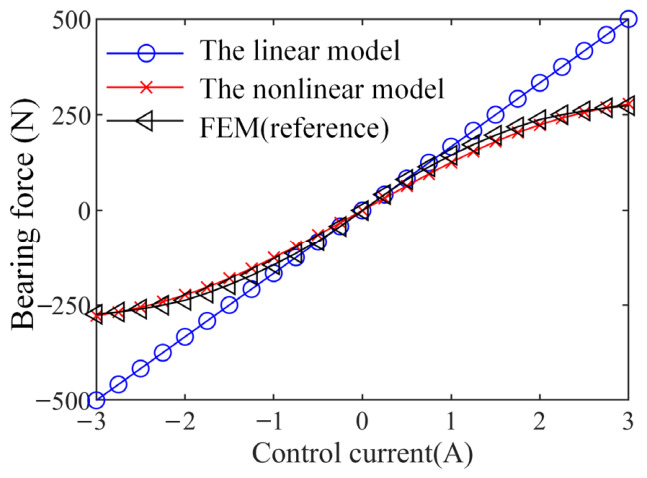
Bearing force–current curves.

Based on Equations (1) to (4), the electromagnetic forces of the AMBs are calculated using both the nonlinear and linear models. At g_0_ = 2.0 mm, the bearing force–current curves from the two models and the finite element method (FEM) are shown in Figure 3. The results indicate that the nonlinear electromagnetic force model agrees well with the FEM results, whereas the linear model exhibits larger deviations at higher control currents.

### 2.2. Dynamical Equations of the MSDS

**Figure 4:**
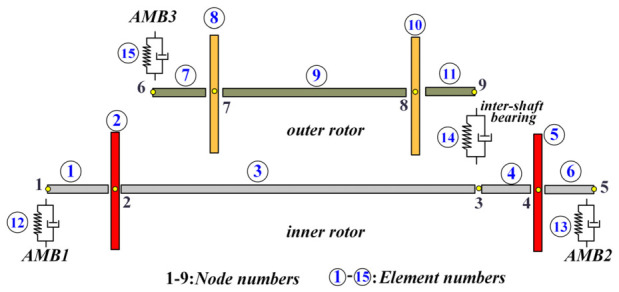
Finite element discretization of the MSDS.

As shown in Figure 4, the MSDS is discretized into fifteen elements and nine nodes using the finite element method. The discretization consists of four disc elements, seven shaft elements, three AMB elements, and one inter-shaft mechanical bearing element. The shaft segments are modeled using Euler–Bernoulli beam elements. The AMBs are represented by equivalent electromagnetic force terms in the equations of motion, whereas the inter-shaft bearing is modeled using equivalent stiffness and damping. According to rotor dynamics theory, the equation of motion of the inner rotor can be written as

(5) $$\left\{\begin{array}{l} M_{i}{\ddot{q}}_{1i}+\omega_{i}J_{i}{\dot{q}}_{2i}+K_{i}q_{1i}=F_{1i},\\ M_{i}{\ddot{q}}_{2i}-\omega_{i}J_{i}{\dot{q}}_{1i}+K_{i}q_{2i}=F_{2i}. \end{array}\right.$$

Here, ***M_i_***, ***J_i_***, and ***K_i_*** are the mass, gyroscopic, and stiffness matrices of the inner rotor system, respectively, and *ω*_i_ represents the rotational speed of the inner rotor. ***F*_1i_** and ***F*****_2i_** are the generalized force vectors of the inner rotor system. The vectors ***q*_1*i*_** and ***q*_2*i*_** denote the generalized displacements of the inner rotor:

(6) $$\left\{\begin{array}{l} q_{1i}={\left\{x_{1}\;\;\;\;\theta_{y1}\;\;x_{2}\;\;\;\;\theta_{y2}\;\cdots \;x_{5}\;\;\;\;\theta_{y5}\right\}}^{T},\\ q_{2i}={\left\{y_{1}\;\;-\theta_{x1}\;\;y_{2}\;\;-\theta_{x2}\;\cdots \;y_{5}\;\;-\theta_{x5}\right\}}^{T}, \end{array}\right.$$

where *x_j_* and *y_j_* (*j* = 1, 2, …, 5) represent the translational displacements of node *j* in the x- and y-directions, respectively; $\theta_{xj}$ and $\theta_{yj}$ (*j* = 1, 2, …, 5) represent the angular displacements of node *j* about the x- and y-axes, respectively.

The generalized force vectors acting on the inner rotor system are expressed as

(7) $$\begin{array}{l} F_{1i}={\left(F_{1ax},\;0,\;0,\;0,\;0,\;0,\;u_{\mathrm{id}}{\omega_{i}}^{2}\sin(\omega_{i}t)+F_{L},\;0,\;F_{2ax\;},\;0\right)}^{T},\\ F_{2i}={\left(F_{1ay},\;0,\;0,\;0,\;0,\;0,\;u_{\mathrm{id}}{\omega_{i}}^{2}\cos(\omega_{i}t),\;0,\;F_{2ay\;},\;0\;\right)}^{T}, \end{array}$$

where *F*_1ax_, *F*_1ay_, *F*_2ax_, and *F*_2ay_ are the nonlinear electromagnetic forces in the *x-* and *y*-directions for AMBs 1 and 2, respectively. $u_{\mathrm{id}}$ denotes the residual unbalance parameter of the inner rotor, and *F*_L_ denotes the external load acting on the corresponding translational degree of freedom. The electromagnetic force components can be obtained from Equations (1)–(4) with the corresponding currents and displacements. The currents are calculated using a PID controller. For example, the control current *i*_1ax_ in the x-direction of AMB 1 is expressed as:

(8) $$i_{1ax}\left(n\right)=K_{P}\cdot \left[x_{\mathrm{ref}}-x_{1}\left(n\right)\right]+K_{D}\cdot \left[\frac{x_{1}\left(n\right)-x_{1}\left(n-1\right)}{\Delta t}\right]+K_{I}\cdot \sum_{k=1}^{n}\left[x_{\mathrm{ref}}-x_{1}\left(k\right)\right],$$

where n is the discrete time-step index; *x*_1_ (*n*) and *x*_1_ (*n* − 1) are the displacements of the node corresponding to AMB 1 in the x-direction at the current and previous time steps, respectively. *x*_ref_ is the reference position in the x-direction of AMB 1 (*x*_ref_ = 0). ∆*t* is the time step. *K*_P_, *K*_D_, and *K*_I_ are the proportional, derivative, and integral gains of the PID controller, respectively.

Similarly, the equation of motion of the outer rotor can be obtained as

(9) $$\left\{\begin{array}{l} M_{o}{\ddot{q}}_{1o}+\omega_{o}J_{o}{\dot{q}}_{2o}+K_{o}q_{1o}=F_{1o},\\ M_{o}{\ddot{q}}_{2o}-\omega_{o}J_{o}{\dot{q}}_{1o}+K_{o}q_{2o}=F_{2o}, \end{array}\right..$$

where ***M_o_***, ***J_o_***, and ***K_o_*** are the mass, gyroscopic, and stiffness matrices of the outer rotor system, respectively; *ω*_o_ represents the rotational speed of the outer rotor. ***F*_1o_** and ***F*****_2o_** are the generalized force vectors of the outer rotor system. The generalized displacements of the outer rotor are defined as

(10) $$\left\{\begin{array}{l} q_{1o}={\left(x_{6},\theta_{6y},x_{7},\theta_{7y},\cdots ,x_{9},\theta_{9y}\right)}^{T},\\ q_{2o}={\left(y_{6},-\theta_{6x},y_{7},-\theta_{7x},\cdots ,y_{9},-\theta_{9x}\right)}^{T}, \end{array}\right..$$

Similarly, *x_j_*, *y_j_*, $\theta_{xj}$ and $\theta_{yj}$ (*j* = 6, 7, …, 9) denote the corresponding translational and angular displacements of node *j* in the outer rotor.

The generalized force vectors acting on the outer rotor system are expressed as

(11) $$\begin{array}{l} F_{1o}={\left(F_{3ax},\;0,\;0,\;0,\;u_{\mathrm{od}}{\omega_{o}}^{2}\sin(\omega_{o}t),\;0,\;0,\;0\right)}^{T},\\ F_{2o}={\left(F_{3ay},\;0,\;0,\;0,\;u_{\mathrm{od}}{\omega_{o}}^{2}\cos(\omega_{o}t),\;0,\;0,\;0\right)}^{T}, \end{array}$$

where *F*_3ax_ and *F*_3ay_ are the nonlinear electromagnetic forces in the *x-* and *y*-directions for AMB 3, respectively, and $u_{\mathrm{od}}$ denotes the residual unbalance parameter of the outer rotor.

The equations of motion for the MSDS are then assembled as:

(12) $$\left[\begin{array}{cc} \begin{array}{cc} M_{i} & \\ & M_{o} \end{array} & 0\\ 0 & \begin{array}{cc} M_{i} & \\ & M_{o} \end{array} \end{array}\right]\left[\begin{array}{c} \begin{array}{c} {\ddot{q}}_{1i}\\ {\ddot{q}}_{1o} \end{array}\\ \begin{array}{c} {\ddot{q}}_{2i}\\ {\ddot{q}}_{2o} \end{array} \end{array}\right]+\left[\begin{array}{cc} 0 & \begin{array}{cc} \omega_{i}J_{i} & \\ & \omega_{o}J_{o} \end{array}\\ \begin{array}{cc} -\omega_{i}J_{i} & \\ & -\omega_{o}J_{o} \end{array} & 0 \end{array}\right]\left[\begin{array}{c} {\dot{q}}_{1i}\\ {\dot{q}}_{1o}\\ {\dot{q}}_{2i}\\ {\dot{q}}_{2o} \end{array}\right]+\left[\begin{array}{cc} \begin{array}{cc} K_{i} & \\ & K_{o} \end{array} & 0\\ 0 & \begin{array}{cc} K_{i} & \\ & K_{o} \end{array} \end{array}\right]\left[\begin{array}{c} q_{1i}\\ q_{1o}\\ q_{2i}\\ q_{2o} \end{array}\right]=\left[\begin{array}{c} F_{1i}\\ F_{1o}\\ F_{2i}\\ F_{2o} \end{array}\right].$$

To account for the inter-shaft bearing, the stiffness and gyroscopic matrices in Equation (12) are modified at the corresponding nodes [12]. The resulting system equations are solved using the Newmark–β method.

The numerical simulations are implemented using a self-developed numerical program. The parameters of the Newmark–β method are set as β = 1/4 and γ = 1/2. In the numerical implementation, the integration step is set to 1 × 10^−5^ s, and the total simulation duration is set to exceed 500 rotor periods for each operating condition. The Poincaré maps were constructed from the steady-state response after the transient response was discarded. Further extension of the simulation duration did not change the number, distribution, or topological features of the Poincaré points, confirming the convergence of the obtained maps. Unless otherwise specified, the initial displacements and velocities of the inner and outer rotors are set to zero. The simulations are conducted under the modelling assumptions and constraints described in Section 2, including the lateral-vibration assumption and the neglect of gravity-induced static deflection.

## 3. Analysis of System Periodic Motion Characteristics

### 3.1. Influence of the Rotational Speed

**Figure 5:**
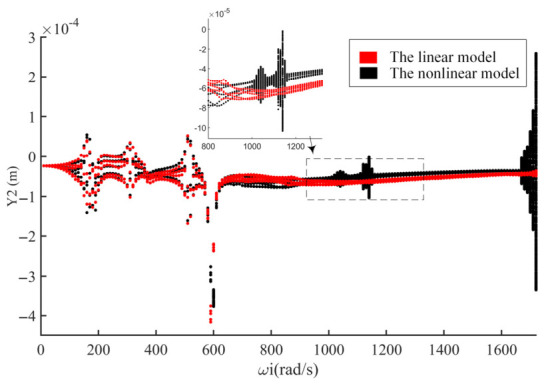
Bifurcation diagram for the vertical response of inner-rotor turbine disc 2.

For *u*_id_ = 8 × 10^−4^ kg·m, *u*_od_ = 6 × 10^−4^ kg·m, *F*_L_ = 200 N, and control parameters *K*_P_ = 40, *K*_D_ = 0.2, and *K*_I_ = 100, the bifurcation diagram of the vibration response of the dual-rotor system for both the linear and nonlinear bearing models is shown in Figure 5. The horizontal axis represents the inner rotor speed *ω*_i_, and the vertical axis represents the vertical displacement of turbine disc 2 (Y_2_).

As shown in Figure 5, in the low-speed region, the systems with both linear and nonlinear bearings exhibit similar periodic responses. However, when *ω*_i_ exceeds 1000 rad/s, the nonlinear bearing system undergoes transitions between periodic and non-periodic states within certain speed intervals, resulting in more complex dynamic behavior than that of the linear case.

**Figure 6:**
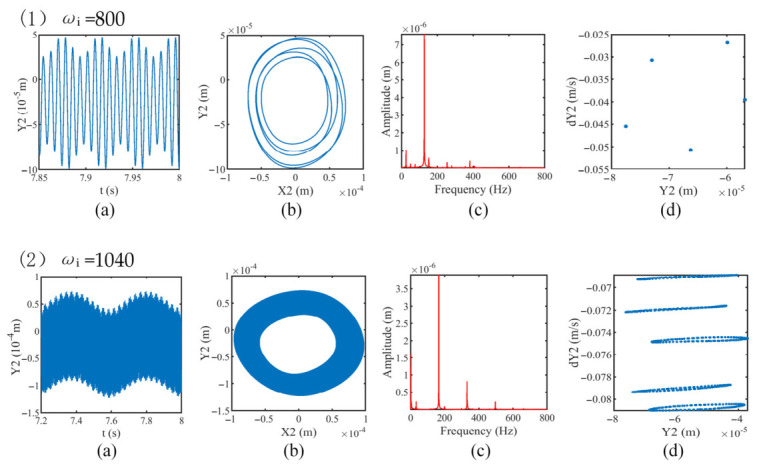

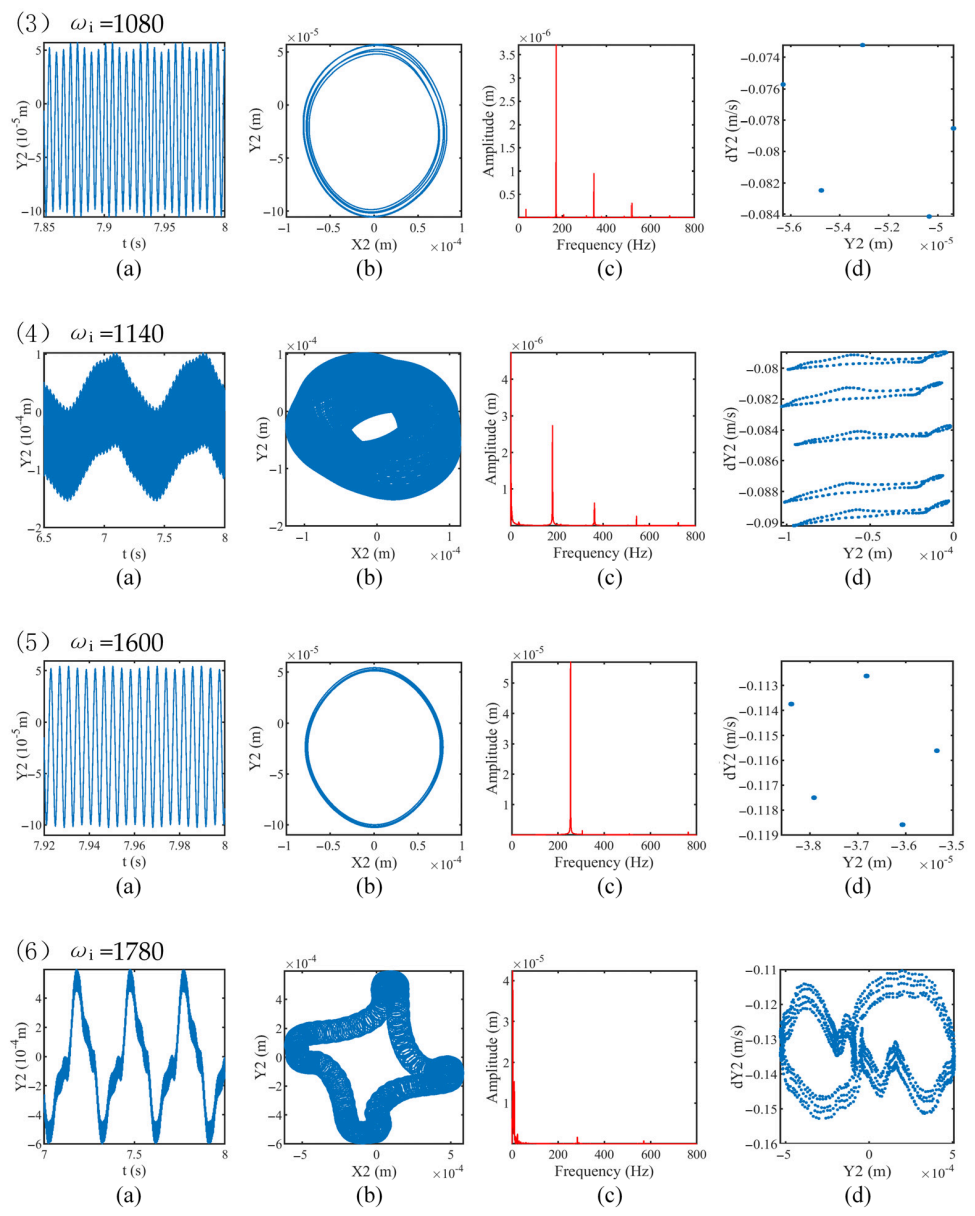
Response of the rotor system at different rotational speeds. (**a**) Time histories; (**b**) Trajectories; (**c**) Spectrum; (**d**) Poincaré maps.

To further examine the influence of AMB nonlinearity on the periodic motion characteristics, the vertical vibration responses of turbine disc 2 at *ω*_i_ = 800, 1040, 1080, 1140, 1600, and 1780 rad/s are analyzed. The corresponding time histories, orbit diagrams, spectra, and Poincaré maps are presented in Figure 6(1–6).

As shown in Figure 5 and Figure 6(1), when *ω*_i_ < 1000 rad/s, the system exhibits a period-5 response. As the rotational speed increases, the first transition from periodic to quasi-periodic motion occurs over the range *ω*_i_ = 1020–1040 rad/s. As shown in Figure 6(2), when *ω*_i_ = 1040 rad/s, the trajectory evolves into a closed annular band, and the Poincaré map displays five closed curves. The spectrum contains not only the fundamental frequency and its combination components but also pronounced low-frequency components, indicating the onset of quasi-periodic motion.

At *ω*_i_ = 1080 rad/s, the system returns to a period-5 state, with a simplified orbit and five discrete points in the Poincaré map. The spectrum is dominated by the rotational-frequency component without significant low-frequency components. As the speed increases to *ω*_i_ = 1140 rad/s, the response again becomes quasi-periodic, as shown in Figure 6(4), indicating a second periodic-to-quasi-periodic transition. The trajectory forms a narrow closed band, and the spectrum is dominated by low-frequency components. The Poincaré map exhibits five twisted and intersecting closed curves, indicating greater dynamical complexity than that observed during the first transition.

Within the range of *ω*_i_ = 1180–1640 rad/s, the system maintains a period-5 response. When the speed reaches *ω*_i_ = 1650 rad/s, quasi-periodic motion reappears. A representative quasi-periodic response at *ω*_i_ = 1780 rad/s is presented in Figure 6(6), indicating a third periodic-to-quasi-periodic transition. The trajectory forms a quadrilateral-like annular band. The Poincaré map exhibits a cross-shaped closed curve composed of hierarchically scattered points, and the low-frequency component in the spectrum becomes dominant, while the rotational-frequency component diminishes significantly. Compared with the case at *ω*_i_ = 1140 rad/s, the response becomes more complex. Further increases in speed lead to stronger nonlinear effects, with trajectories gradually evolving into scattered patterns.

**Figure 7:**
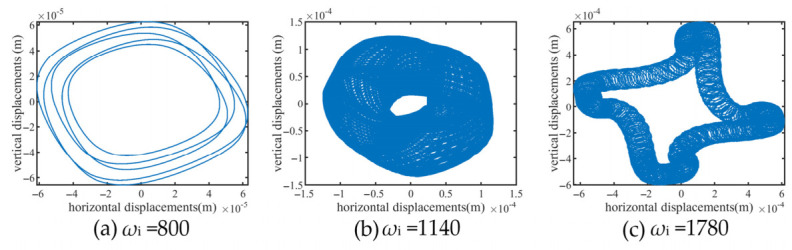
Trajectories at AMB 2.

**Figure 8:**
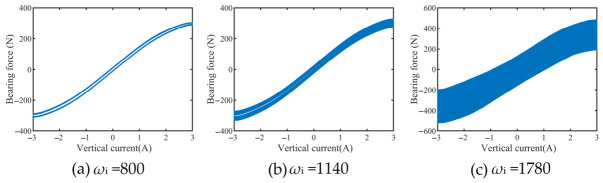
Bearing force–current curves at AMB 2.

Further analyses of the trajectories and force–current characteristics of AMB 2 at different rotational speeds are presented in Figure 7 and Figure 8.

As shown in Figure 6 and Figure 7, the response at AMB 2 exhibits periodic motion characteristics similar to those observed at turbine disc 2. The amplitude of the rotor-shaft displacement varies with rotational speed. Compared with the period-5 motion at *ω*_i_ = 800 rad/s, the displacement amplitude is larger during quasi-periodic motion (*ω*_i_ = 1140, 1780 rad/s). Correspondingly, the force–current characteristics of AMB 2 spread over a wider range, indicating enhanced nonlinear support behavior, as illustrated in Figure 8.

These repeated transitions between periodic and quasi-periodic motions are associated primarily with the combined influence of nonlinear bearing characteristics and the speed-dependent dynamic response of the dual-rotor system. Their sensitivity to variations in initial stiffness and damping is further examined in the following sections.

### 3.2. Influence of Initial Stiffness

The stiffness and damping of AMBs play a critical role in determining the dynamic characteristics of the system. In AMB design, variations in control laws and parameters lead to different initial stiffness and damping values when the material properties, structural parameters, and operating positions are fixed. Under PID control, the initial stiffness (*K*_0_) and damping (*C*_0_) of the AMBs can be expressed as

(13) $$\left\{\begin{array}{l} K_{0}=k_{i}A_{a}A_{s}K_{P}-k_{x},\\ C_{0}=k_{i}A_{a}A_{s}K_{D}. \end{array}\right.$$

**Figure 9:**
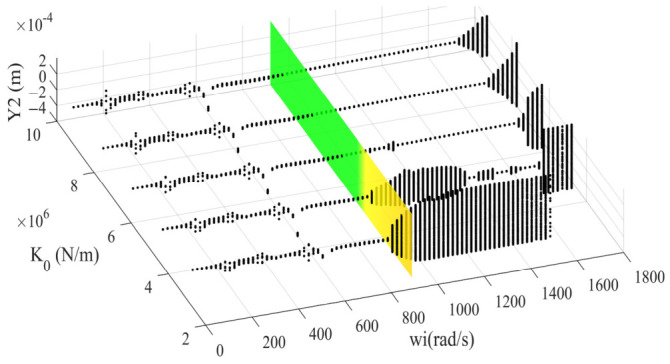
Bifurcation diagram of the vibration response of disc 2 for different *K*_0_. (The colored section corresponds to the selected section further analyzed in Figure 10).

The stiffness considered here neglects nonlinear effects such as flux leakage and magnetic saturation during operation. To investigate the influence of initial stiffness on the periodic motion characteristics of the system with nonlinear support, a bifurcation diagram of the vertical response of the rotor disc is constructed by varying *K*_P_, as shown in Figure 9.

**Figure 10:**
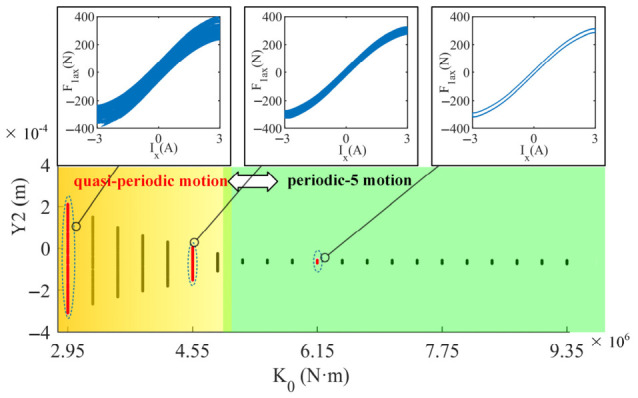
Bearing force–current curves at AMB 2 with different *K*_0_.

As shown in Figure 9, the periodic motion characteristics of the system vary with different initial stiffness values *K*_0_. In the low-speed region, the influence of *K*_0_ is negligible; therefore, the analysis focuses on the higher-speed range of *ω*_i_ = 800–1700 rad/s:

For *K*_0_ = 9.35 × 10^6^ N/m and 7.75 × 10^6^ N/m, the system exhibits stable period-5 motion, which remains unchanged within the investigated stiffness range. When *K*_0_ = 6.15 × 10^6^ N/m, quasi-periodic motion emerges within *ω*_i_ ∈ [1020, 1060] and [1120, 1180] rad/s, indicating increased dynamic complexity.

For *K*_0_ = 4.55 × 10^6^ N/m, quasi-periodic motion occurs three times within *ω*_i_ ∈ [920, 1320], [1380, 1440], and [1520, 1540] rad/s, with the first onset at *ω*_i_ = 920 rad/s. At the lowest stiffness considered, *K*_0_ = 2.95 × 10^6^ N/m, quasi-periodic motion initiates at *ω*_i_ > 880 rad/s and persists until the trajectory diverges at *ω*_i_ = 1560 rad/s.

In summary, within the investigated parameter range, decreasing initial stiffness is associated with an earlier onset and a higher occurrence frequency of quasi-periodic motion, suggesting that lower initial stiffness tends to strengthen the nonlinear dynamic behavior of the system.

To further illustrate this effect, the system responses and force–current characteristics of AMB 2 at *ω*_i_ = 920 rad/s under different stiffness conditions are presented in Figure 10. As the stiffness increases, the system transitions from quasi-periodic motion to stable periodic motion, demonstrating that the control system can actively regulate the system from a quasi-periodic state to a periodic state.

Furthermore, during periodic motion, the fluctuation amplitude of the force–current curve is relatively small, whereas it increases significantly during quasi-periodic motion. This behavior reflects the emergence of a multi-valued force–current relationship and indicates stronger nonlinear support characteristics, thereby providing an effective indicator of the complexity of the system dynamics.

### 3.3. Influence of Initial Damping

**Figure 11:**
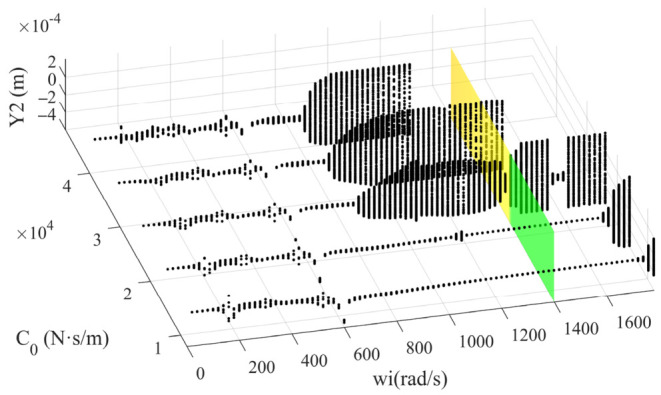
Bifurcation diagram of the vibration response of disc 2 for different *C*_0_. (The colored section corresponds to the selected section further analyzed in Figure 12).

Similarly, the influence of initial damping on the periodic motion characteristics of the MSDS is investigated by varying the control parameter *K*_D_, as shown in Figure 11. At low rotational speeds, the system exhibits stable period-5 motion regardless of the damping value. Consistent with the influence of initial stiffness, the effect of initial damping on the system’s motion characteristics is primarily observed in the medium- and high-speed ranges. Detailed observations are as follows:

**Figure 12:**
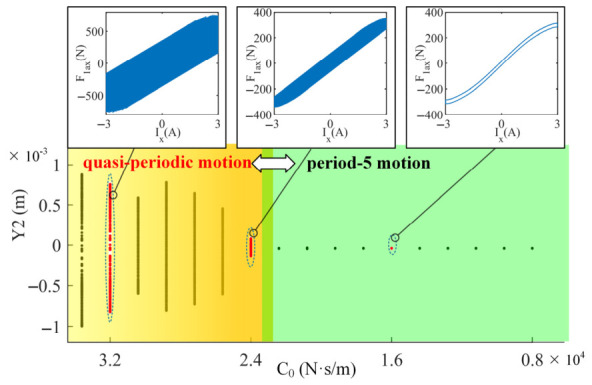
Bearing force–current curves at AMB 2 with different *C*_0_.

For *C*_0_ = 8 × 10^3^ N·s/m, the system maintains period-5 motion within *ω*_i_ ∈ [0, 1760] rad/s. Beyond this range, further increases in rotational speed induce quasi-periodic motion, eventually leading to instability.

When the damping increases to *C*_0_ = 1.6 × 10^4^ N·s/m and 2.4 × 10^4^ N·s/m, quasi-periodic motion appears within the originally stable periodic domain (*ω*_i_ ∈ [0, 1760] rad/s), alternating with periodic motion in the range *ω*_i_ ∈ [840, 1760] rad/s. Increasing damping advances the onset of quasi-periodic motion from *ω*_i_ = 1020 rad/s to 840 rad/s and extends its duration.

With further increases in damping (*C*_0_ = 3.2 × 10^4^ and 4.0 × 10^4^ N·s/m), the first occurrence of quasi-periodic motion shifts to *ω*_i_ = 800 rad/s. As the rotational speed increases, the system no longer returns to periodic motion after the onset of quasi-periodicity and eventually evolves toward instability.

In summary, within the investigated parameter range, increasing initial damping tends to advance the onset of quasi-periodic motion and expand its occurrence interval, suggesting a stronger nonlinear dynamic response of the system.

Furthermore, at *ω*_i_ = 1400 rad/s, the force–current characteristics of AMB 2 under different damping conditions are illustrated in Figure 12. As the damping decreases, the system transitions from quasi-periodic to periodic motion, accompanied by corresponding changes in the force–current curves. These variations demonstrate that the force–current curve serves as an effective indicator of the system’s motion state.

### 3.4. Influence of Control Parameter Adjustment

To further investigate the regulating effect of the control system on the periodic motion characteristics of the magnetic bearing–rotor system, time-varying operating conditions are considered. Variations in external conditions are introduced, and the control parameters are adjusted online to examine the evolution of motion states.

Based on Equation (13) and the parametric results in Section 3.2 and Section 3.3, the PID parameters were selected according to their effects on the equivalent support stiffness and damping. Since a lower initial stiffness *K*_0_ was found to advance the onset and increase the occurrence of quasi-periodic responses, *K*_P_ was increased in the present adjustment to raise the equivalent support stiffness and move the system toward a more stable periodic-motion region. The derivative gain *K*_D_ mainly determines the initial damping *C*_0_; however, excessive damping may also advance the onset of quasi-periodic responses in the present nonlinear electromechanically coupled system. Therefore, *K*_D_ should be kept within an appropriate range rather than simply increased. The integral gain *K*_I_ is mainly used to reduce the steady-state displacement error and was kept unchanged in this adjustment to avoid introducing additional low-frequency control action.

**Figure 13:**
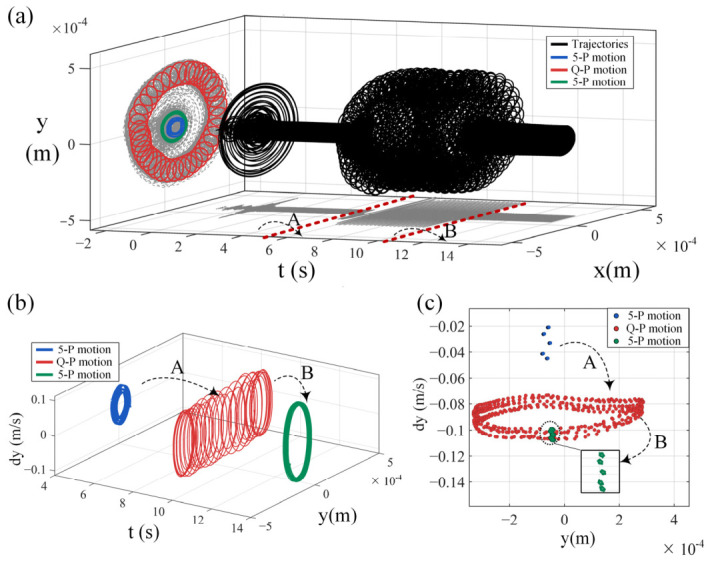
Evolution of system motion under control-parameter adjustment. (**a**) 3D trajectories; (**b**) Phase portraits; (**c**) Poincaré maps. (A: after operating-condition change; B: after PID switching).

First, a set of stable levitation control parameters (PID1, *K*_P_ = 40, *K*_D_ = 0.3, *K*_I_ = 100) is selected to ensure that the system operates in a periodic motion state. At *t* = 4 s, a change in operating conditions is introduced by increasing the rotational speed from 800 rad/s to 1200 rad/s. Subsequently, at *t* = 8 s, the control parameters are switched to PID2 (*K*_P_ = 120, *K*_D_ = 0.1, *K*_I_ = 100) according to this parameter-selection principle. During this process, the phase portraits, Poincaré maps, and rotor center trajectories are recorded, as shown in Figure 13.

The results show that, following the change in operating conditions at *t* = 4 s, the system evolves from an initial period-5 motion to a quasi-periodic state. The rotor center trajectory expands significantly, accompanied by intensified vibration response and increased motion complexity. After the control parameters are adjusted at *t* = 8 s, the system undergoes a brief transient process and subsequently returns to a period-5 state. Meanwhile, the trajectory range of the rotor center decreases, indicating an improvement in system stability.

These results demonstrate that the tunable support characteristics of magnetic bearings can be used to regulate system motion states. By appropriately adjusting control parameters, quasi-periodic motion can be suppressed and periodic motion can be restored, providing a feasible and effective approach for maintaining desired dynamic states under complex operating conditions.

## 4. Experimental Validation

To validate the proposed nonlinear dynamic model and to investigate the influence of system parameters on periodic motion characteristics, two sets of experiments were conducted on a custom-built MSDS test rig:

(1) Stiffness identification experiments, including current-stiffness and displacement-stiffness measurements of the AMBs, were conducted to validate the nonlinear electromagnetic force model;
(2) Unbalance response experiments under different parameter conditions were performed to verify the evolution of periodic responses and to assess the model’s ability to reproduce the observed dynamic behavior.

**Figure 14:**
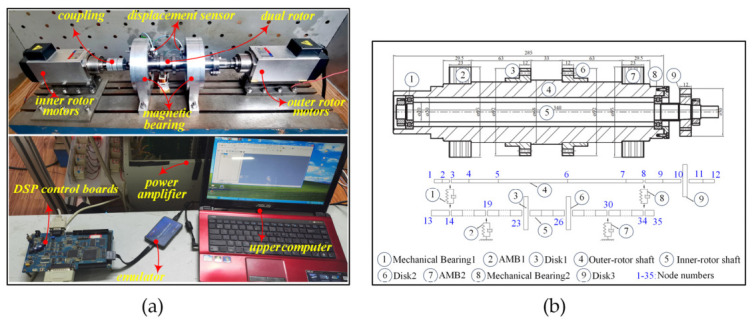
Schematic diagram of the MSDS test rig. (**a**) MSDS test rig; (**b**) MSDS structure and finite-element model.

The experimental setup is shown in Figure 14. The mechanical system consists of dual rotors, magnetic bearings, and driving motors. The outer rotor is supported by two AMBs, while the inner and outer rotors are interconnected via inter-shaft bearings and driven by motors at both ends. Eddy current displacement sensors are installed in both horizontal and vertical directions to measure rotor displacements. The measured signals are fed into the controller to generate control voltages, which are subsequently amplified into control currents by the power amplifier and supplied to the magnetic bearings. Unbalance excitation is introduced by attaching bolt assemblies of different masses to the uniformly distributed holes on the rotor discs. In the stiffness identification experiments, static loads are applied using suspended weights.

### 4.1. Stiffness Identification Experiments

**Figure 15:**
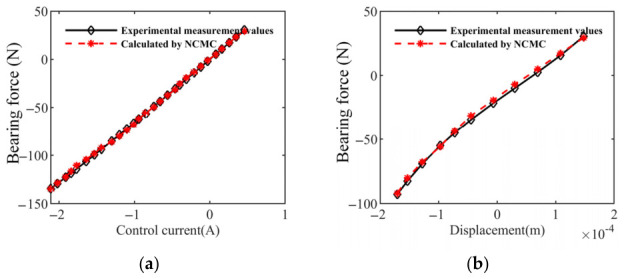
Current/displacement stiffness of the AMB 1. (**a**) Current stiffness; (**b**) Displacement stiffness.

The current stiffness of AMB 1 is identified by applying incremental static loads to the levitated rotor at a stable equilibrium position. Loads are applied in increments of 2 kg starting from 0 kg, and after each loading step reaches a steady state, the corresponding coil current is recorded. The relationship between the applied load and the measured current is presented in Figure 15a.

The displacement stiffness of AMB 1 is identified using a similar loading procedure, while maintaining a constant control current. After each loading step reaches a steady state, the corresponding suspension displacement is adjusted and recorded, together with the applied force. The relationship between force and displacement is obtained and presented in Figure 15b.

Based on the structural parameters of AMB 1, the NMCM is employed to establish the electromagnetic force model. The current and displacement stiffness values are calculated and compared with the experimental results, as shown in Figure 15.

The model predictions show good agreement with the experimental results, thereby confirming the validity of the established model. As the suspension displacement increases, the displacement stiffness exhibits pronounced nonlinear characteristics, which are accurately captured by the proposed model.

### 4.2. Unbalance Response Experiments

**Figure 16:**
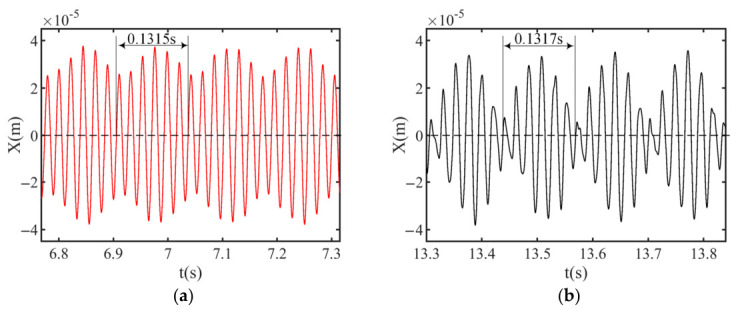
Time histories of the horizontal vibration response at AMB 2. (**a**) Numerical result; (**b**) Experimental result.

Under the operating conditions of *ω*_i_ = 38 Hz, *r_w_* = 1.2, *u*_id_ = 7.41 × 10^−5^ kg·m, and *u*_od_ = 2.548 × 10^−4^ kg·m, the unbalance responses of the MSDS were obtained numerically and experimentally, as shown in Figure 16. Owing to the small difference between the rotational speeds of the inner and outer rotors, a pronounced beat vibration is observed in the system response. The numerical predictions agree well with the experimental results in terms of both the maximum vibration amplitude and the beat period (t ≈ 0.131 s). Minor discrepancies between the numerical and experimental results are likely attributable to experimental uncertainties, such as electrical interference, coupling-induced stiffness, and assembly tolerances. Electrical interference may introduce local fluctuations into the displacement and current signals, whereas coupling-induced stiffness and assembly tolerances may slightly alter the effective boundary conditions of the rotor system. These factors can cause minor deviations in vibration amplitude and phase, but do not change the dominant beat and periodic vibration characteristics. Therefore, the proposed model is applicable to predicting the main periodic responses of the MSDS under stable levitation conditions. However, random noise, detailed coupling flexibility, and assembly errors are not explicitly modeled, and experimental calibration is still required for high-precision engineering prediction.

**Figure 17:**
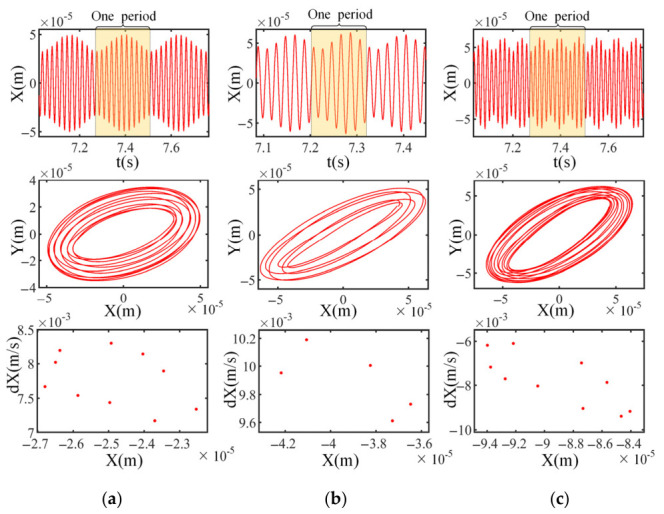
Time histories and trajectories of the rotor under different speed ratios (numerical results). (**a**) *r_w_* = 1.1. (**b**) *r_w_* = 1.2. (**c**) *r_w_* = 1.3.

**Figure 18:**
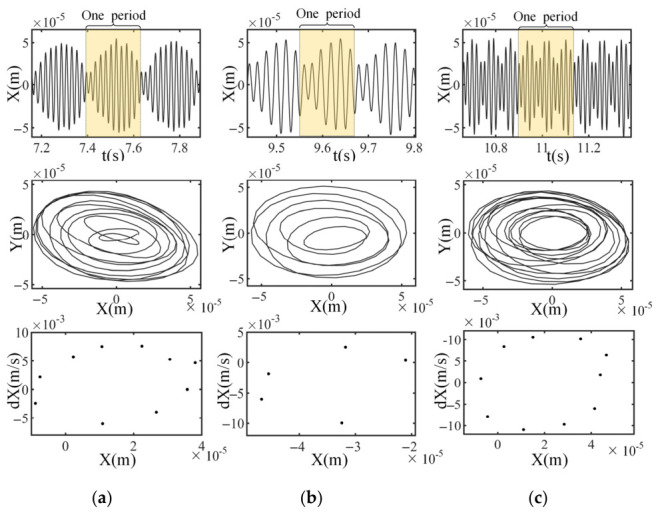
Time histories and trajectories of the rotor under different speed ratios (experimental results). (**a**) *r_w_* = 1.1. (**b**) *r_w_* = 1.2. (**c**) *r_w_* = 1.3.

To further validate the proposed dynamic model and examine the influence of operating parameters on the periodic responses, numerical and experimental analyses of the MSDS were carried out under different speed ratios. Specifically, the system was tested at *ω*_i_ = 42 Hz, *u*_id_ = 7.41 × 10^−5^ kg·m, and *u*_od_ = 2.548 × 10^−4^ kg·m, with rotational speed ratios *r_w_* = 1.1, 1.2, and 1.3, as shown in Figure 17 and Figure 18.

At *r_w_* = 1.1, the system exhibits a periodic motion with a period of 0.238 s, corresponding to 11 vibration peaks and a rotor trajectory forming 11 distinct closed loops. At *r_w_* = 1.2, the motion period decreases to 0.119 s, with the number of peaks reducing to six and the trajectory simplifying to six loops. When *r_w_* = 1.3, the period increases again, accompanied by an increase in the number of peaks and a more complex trajectory. The corresponding Poincaré maps reveal 10, 5, and 10 points for *r_w_* = 1.1, 1.2, and 1.3, respectively, reflecting the parameter-dependent variations in periodic motion.

The observed results indicate that the system’s nonlinear periodic behavior is highly sensitive to variations in the operating parameters. Additionally, the numerical simulations exhibit good agreement with the experimental observations, thereby confirming the validity of the proposed dynamic model for capturing the system response under different operating conditions.

## 5. Conclusions

In this paper, a nonlinear dynamic model of the MSDS with mass unbalance was established based on a nonlinear bearing model considering flux leakage and magnetic saturation effects, and its validity was verified through numerical–experimental comparison. The main conclusions are summarized as follows:

(1) The system exhibits period-5 and quasi-periodic motions induced by bearing nonlinearities, and transitions between these states occur in the high-speed region under certain parameter conditions.
(2) Compared with period-5 motion, quasi-periodic motion is characterized by more complex rotor orbits and larger vibration amplitudes. In addition, the force–current characteristics broaden into a wider band, and this variation can serve as an indicator of the system’s motion state.
(3) Within the investigated parameter range, the influences of initial stiffness *K*_0_ and initial damping *C*_0_ are more pronounced in the high-speed region. Lower *K*_0_ and higher *C*_0_ are associated with an earlier onset and a higher occurrence frequency of quasi-periodic motion, whereas appropriate *K*_0_ and *C*_0_ improve system stability.
(4) When operating conditions change, online tuning of the control parameters can drive the system from quasi-periodic motion back to a stable period-5 state. This demonstrates that the tunable support characteristics of AMBs enable active regulation of system motion states and provide an effective control approach for maintaining desired dynamic states under time-varying operating conditions.

## Figures and Tables

**Table 1:** Main parameters of the MSDS.

Physical Parameter	Value	Physical Parameter	Value
Length of inner rotor (m)	0.706	Outside and inside radii of disc 2 (m)	0.125, 0.0125
Length of outer rotor (m)	0.5011	Outside and inside radii of disc 3 (m)	0.125, 0.02
Outside and inside radii of inner rotor (m)	0.0125, 0.0075	Outside and inside radii of disc 4 (m)	0.125, 0.02
Outside and inside radii of outer rotor (m)	0.02, 0.015	Thickness of disc (m)	0.0273
Density of rotating shaft (kg/m^3^)	7850	Density of disc (kg/m^3^)	7928.56
Elastic modulus (Pa)	2.1 × 10^11^	Residual unbalance of disc 2, *u*_id_ (kg·m)	8 × 10^−4^
The nominal air-gap length, g_0_ (m)	2 × 10^−3^	Residual unbalance of disc 4, *u*_od_ (kg·m)	6 × 10^−4^
Poisson’s ratio	0.3	Inter-shaft bearing stiffness (N/m)	1 × 10^6^
Outside and inside radii of disc 1 (m)	0.125, 0.0125	Inter-shaft bearing damping (N·s/m)	100

## Data Availability

The raw data supporting the conclusions of this article will be made available by the authors on request.

## Appendix A. Nomenclature

**Table A1:** Nomenclature of variables and parameters used in this study.

Symbol/Parameter	Description
**Magnetic Bearing and Electromagnetic-Force Parameters**	
*I_b_*	Bias current of the AMB.
*I_c_*	Control current of the AMB.
*g*_0_	Nominal air-gap length of the AMB.
*A_s_*	Displacement sensor gain.
*A_a_*	Power amplifier gain.
*R_g_*_1_, *R_g_*_2_	Upper air-gap reluctances associated with Φ1.
*R_g_*_3_, *R_g_*_4_	Lower air-gap reluctances associated with Φ3.
*R_y_*	Stator-yoke reluctance.
*R_p_*	Stator-pole reluctance.
*R_r_*	Rotor reluctance.
*F*_1_ *–F*_4_	Magnetomotive force sources of the four poles in the EMC.
*α*	Angle between the centerlines of the pole and the pole pair.
*μ*_0_	Permeability of free space.
*A_p_*	Cross-sectional area of the stator poles.
*N*	Number of coil turns.
*Φ*_1_	Magnetic flux passing through the upper air gaps between the pole pair and the rotor.
*Φ*_3_	Magnetic flux passing through the lower air gaps between the pole pair and the rotor.
*k_x_*	Displacement stiffness of the AMB in the linear electromagnetic-force model.
*k_i_*	Current stiffness of the AMB in the linear electromagnetic-force model.
*F*_1*ax*_, *F*_1*ay*_	Nonlinear electromagnetic force components of AMB 1 in the x- and y-directions.
*F*_2*ax*_, *F*_2*ay*_	Nonlinear electromagnetic force components of AMB 2 in the x- and y-directions.
*F*_3*ax*_, *F*_3*ay*_	Nonlinear electromagnetic force components of AMB 3 in the x- and y-directions.
**Rotor-Dynamic Variables and Force Vectors**	
*M_i_*, *J_i_*, *K_i_*	Mass, gyroscopic, and stiffness matrices of the inner rotor system.
*M_o_*, *J_o_*, *K_o_*	Mass, gyroscopic, and stiffness matrices of the outer rotor system.
*ω_i_*	Rotational speed of the inner rotor.
*ω_o_*	Rotational speed of the outer rotor.
*q*_1*i*_, *q*_2*i*_	Generalized displacement vectors of the inner rotor.
*q*_1*o*_, *q*_2*o*_	Generalized displacement vectors of the outer rotor.
*F*_1*i*_, *F*_2*i*_	Generalized force vectors of the inner rotor system.
*F*_1*o*_, *F*_2*o*_	Generalized force vectors of the outer rotor system.
*x_j_*, *y_j_*	Translational displacements of node j in the x- and y-directions.
*θ_xj_*, *θ_yj_*	Angular displacements of node j about the x- and y-axes.
*u*_id_	Residual unbalance parameter of the inner rotor.
*u*_od_	Residual unbalance parameter of the outer rotor.
*r_w_*	Rotational speed ratio between the inner and outer rotors.
**Control and numerical-integration parameters**	
*i*_1*ax*_	Control current in the x-direction of AMB 1.
*x*_1_ (*n*)	Displacement of the node corresponding to AMB 1 in the x-direction at the current time step.
*x*_1_(*n* − 1)	Displacement of the node corresponding to AMB 1 in the x-direction at the previous time step.
*x_ref_*	Reference position in the x-direction of AMB 1.
*n*	Discrete time-step index.
*Δt*	Time step used in the numerical integration.
*K_P_*, *K_D_*, *K_I_*	Proportional, derivative, and integral gains of the PID controller.
*K*_0_	Initial stiffness of the AMB under PID control.
*C*_0_	Initial damping of the AMB under PID control.
*β*, *γ*	Parameters of the Newmark–β integration method.
